# Early detection of distinct language development in infants of mothers with severe mental illness using fNIRS

**DOI:** 10.1038/s44271-026-00475-y

**Published:** 2026-06-15

**Authors:** Alessandra Piatti, Holly Hope, Matthias Pierce, Kathryn Abel

**Affiliations:** 1 Centre for Women’s Mental Health, Division of Psychology and Mental Health, Faculty of Biology, Medicine and Health, University of Manchester, Manchester, UK; 2 Greater Manchester Mental Health NHS Foundation Trust, Manchester, UK; 3 Present Address: MANIBUS Lab, Psychology Department, University of Turin, Turin, Italy

**Keywords:** Risk factors, Language

## Abstract

Infants of mothers with severe mental illness are at elevated risk of neurodevelopmental difficulties. This study used functional near-infrared spectroscopy (fNIRS) to compare 8–10-month-old infants exposed to severe maternal mental illness (*n* = 30) and unexposed controls (*n* = 30) during two experiments assessing brain activation to voice versus non-voice sounds and to sentences with angry, happy, or neutral prosody. Exposed infants showed atypical right-temporal activation, with stronger activation to non-voice than voice stimuli, unlike controls. Socioeconomic status was positively correlated with activation to non-voice and to sentences, independently of emotional prosody. Further analysis indicated that brain response to non-voice stimuli mediated the relation between socioeconomic status and activation to sentences. These findings reveal distinct neural trajectories in infants exposed to severe maternal mental illness, detectable within the first year of life, and highlight socioeconomic background as a key factor in early brain development.

## Abstract (web summary)

Brain responses to voice differ at 9 months in infants born to mothers with severe mental illness. Socioeconomic status also relates to how infants process sounds, suggesting multiple early factors associated with language development.

## Introduction

The developmental pathway of children at elevated likelihood for neurodevelopmental disorders has been studied extensively^1–3^, principally with the scope of early detection and to enable early intervention. Research on the behavioural phenotype of children who go on to have a developmental disorder has identified early signs which can be detected by parents or caregivers^4,5^ and trigger further monitoring and possibly support^6,7^. Neuroimaging techniques, on the other hand, have identified broader developmental atypicalities in cohorts defined by an elevated likelihood of neurodevelopmental disorder^8^, and a more specific divergence from typical development in subgroups of high-likelihood children who later go on to develop a full-blown disorder^9^.

The early developmental pathway of children and adolescents whose parents have mental illness has however been relatively understudied, despite the increasing prevalence of exposure to parental mental illness in children^10^ and its causal association with neurodevelopmental disorders^11,12^.

Admittedly, it has been shown that below-average verbal IQ is more prevalent among infants exposed to parental mental illness than in their unexposed peers, either independently or in association with other neurodevelopmental symptomatology^13,14^. However, language delays are often not noticed by caregivers before pre-school age, and even psychometric instruments can only reliably detect atypical language development starting from the second or third year of life^15,16^ Findings on language delay in infants born to parents with mental illness, which may in turn be a sign of neurodevelopmental disorder or elevated likelihood for mental illness, trigger the question of whether earlier signs of atypical language development might be observed using neuroimaging techniques. In particular, functional near-infrared spectroscopy (fNIRS) has shown potential in recent years to provide insights into the neural developmental pathway of elevated-likelihood cohorts^17,18^, and possibly to offer candidate biomarkers to support, together with behavioural assessments, the early detection of atypicalities at the individual level, which in turn could facilitate access to early intervention.

In this study, we used fNIRS to investigate neural processing of language in 9-month-old infants born to mothers with severe mental illness and in control infants with a typical likelihood for language delay and neurodevelopmental disorders. Severe mental illness primarily refers to psychotic disorders, and includes severe cases of mood and anxiety disorders, which require inpatient admission. Neurodevelopmental disorders and adverse mental health outcome are more common in infants born to parents with severe mental illness^19,20^ than those born to parents with common mental illness, particularly if the affected parent is the mother^21^ and independently from in utero exposure^12^. Data on recognised factors contributing to language atypicalities were also collected, to control for possible confounds linked to infant sex, socioeconomic status, birthweight, minority background, familial risk for neurodevelopmental disorder other than the one associated with severe maternal mental illness, i.e. linked to maternal learning disability or paternal mental illness.

Using well-validated stimuli^22–24^, we tested the hypotheses that exposed infants show significant differences in language development as measured by fNIRS at 9 months compared to control infants, independently from key maternal variables, and that the joint effect of severe maternal mental illness and other factors accentuates atypicalities of neural activity.

More specifically, in Experiment 1 we compared voice and non-voice processing, and in Experiment 2 we investigated the neural processing of emotional intonation (prosody) in semantically neutral stimuli. In exposed infants, we expected attenuated responses to voice and diminished discrimination of emotional prosody compared to control, independently from additional factors contributing to increase the likelihood of atypical language processing. Additionally, we expected other factors associated with elevated likelihood for language delay and neurodevelopmental disorders to contribute to atypical neural processing of stimuli in the same direction as severe maternal mental illness.

## Methods

This study was partially pre-registered on ClinicalTrials.gov (Identifier: NCT04350294) in April 2020. The pre-registration included the study rationale, research questions, and stimulus descriptions; the analysis plan was not pre-registered.

### Participants

Thirty infants exposed to severe maternal mental illness and 30 unexposed infants contributed fNIRS data to both experiments in this study. fNIRS data collected from 15 additional children (7 exposed, 8 unexposed) were discarded due to insufficient quality or quantity of collected data as detailed in Section “Data collection and processing” and excluded from all analyses. Infants were between 8 and 10 months old at the time of the visit (exposed: *M* = 8.98, *SD* = 0.81; unexposed: *M* = 9.07, *SD* = 1.12). This age range was chosen to capture language processing peculiarities at a preverbal stage in the exposed cohort, in line with other prospective studies in infants with high likelihood for neurodevelopmental disorders^8,9,25^.

At follow-up (12 months), infants’ verbal and non-verbal IQ was assessed using the Bayley III test^26^. Twenty-one exposed and 26 unexposed infants with valid fNIRS data at 9 months also provided IQ data at 12 months.

Mothers with severe mental illness were recruited from 16 NHS trusts participating in the research project. Medical records were used for pre-screening and to extract diagnostic data. Control mothers were recruited via social media advertising, were referred to online participant information, completed anonymous pre-screening and, if eligible, were referred to the enroling procedure. All mothers were cis-women. The study was approved through North West–Greater Manchester West Research Ethics Committee (17/NW/0074) and Health Research Authority (212715). Mothers gave informed consent.

The inclusion criterion for the mothers with severe mental illness was a current diagnosis of psychotic disorder (codes F20 – F29 in the ICD-10^27^, 7 mothers, 6 with comorbid mood disorder) or mood disorder (F30–F39, 26 mothers, 6 with comorbid psychotic disorder). Diagnoses of anxiety disorders (F40–F48) without comorbid psychotic or mood disorder were considered severe mental illness based on the patient’s history, as assessed by the responsible healthcare provider (3 mothers). Data on the symptom severity of mental illness in mothers with mental illness were collected using the Brief Psychiatric Rating Scale (BPRS)^28^. Data on current or previous medication use (from pregnancy onwards) in mothers with mental illness was retrieved from medical records. In this sample, all mothers assumed psychiatric medication. Sertraline was used by 22 mothers, quetiapine by 10, citalopram by 8, aripiprazole by 7, and fluoxetine by 6 mothers. Five mothers or less assumed diazepam, lorazepam, haloperidol or olanzapine.

Mother-infant pairs were not enrolled into the study if: the infant had poor general health, including a congenital condition; the mother was younger than 16; the mother did not have parental rights on the child; the mother did not have sufficient capacity to consent. For the unexposed group, mother-child pairs were excluded if there was a diagnosis or treatment for a mental illness, or mental illness symptomatology, in the 5 years prior to enrolment. Screening positive (≥11 out of 20 in either subscale) for anxiety or depression based on the Hospital Anxiety and Depression Scale (HADS)^29^ was also an exclusion criterion for control mothers. Finally, mother-infant pairs in the exposed group were not enrolled if the mother had a primary diagnosis of bulimia or anorexia nervosa, alcohol or substance dependence, autism spectrum disorder, intellectual disability, or personality disorder, given the known and specific relation between these maternal factors and child development.

In addition to data on psychiatric symptomatology, information on socioeconomic and demographic background was recorded (Table 1).

**Table 1:** Description of the final samples of exposed and non-exposed infants

	Exposed (*n* = 30)	Unexposed (*n* = 30)
1. Infants’ sex		
Girls	14	9
Boys	16	21
2. Infants’ birth order	1.63 (0.85)	1.50 (0.77)
3. Maternal minority background	8	8
4. Maternal learning disability	4	3
5. Paternal mental illness^b^	9	2
6. Infants’ age (months)^a^	8.98 (0.81)	9.07 (1.12)
7. Maternal age at childbirth (years)^b^	30.65 (4.97)	32.99 (4.05)
8. SES^a,c^	4.00 (2.37)	5.00 (1.50)
9. Infants’ gestational age at birth (weeks)^b^	38.62 (1.86)	39.65 (1.42)
10. Birthweight (grams)^b^	2889 (593)	3263 (465)
11. Mothers’ HADS score^d^ Anxiety subscale^d^ Depression subscale^d^	20.36 (6.82) 11.96 (4.19) 8.40 (3.48)	9.13 (4.91) 5.73 (2.85) 3.40 (2.56)
12. Mothers’ BPRS score	40.10 (11.52)	n.a.

Data were collected from 30 infants exposed to severe maternal mental illness and 30 controls. All mothers were cis-women. For variables 1–4 the statistic reported is the count of cases for each group. For variables 5–11, the mean and *SD* are shown. *HADS* Hospital Anxiety and Depression Scale (Zigmond & Snaith, 1983). *BPRS* Brief Psychiatric Rating Scale (Overall & Gorham, 1962).

^a^The normality assumption was violated in at least one group: the reported measures are median and interquartile range.

^b^Group difference (bootstrap based *t*test) is significant for *α* = 0.05.

^c^Group difference (bootstrap based *t*-test) is significant for *α* = 0.005.

^d^Group difference (bootstrap based *t*test) is significant for *α* = 0.001.

Socioeconomic status was calculated based on household income, ranked 1–6, and maternal education, ranked 1–7. For the sake of dimension reduction, the two indicators were aggregated by computing the mean rank. Maternal education was ranked 1–7, corresponding to the following qualifications or their equivalent: 1 = no formal education degree; 2 = GCSE, 3: A-level; 4 = Higher national certificate/diploma; 5 = Bachelor’s degree; 6 = Master’s degree; 7 = PhD degree. Family income range was ranked 1–6: 1 = £ 0–5,475; 2 = 5476 – 14,999; 3 = 15,000–30,999; 4 = 31,000–45,999; 5 = 46,000–60,999; 6 = 61,000 or higher.

Maternal learning disability was coded as a dichotomous variable, based on self-report. Similarly, maternal minority background was coded dichotomously, based on whether the mother identified as belonging to a racial or ethnic minority, or on her migration background. Dichotomous data on paternal mental illness and infant sex were recorded based on maternal report.

Infant sex distribution was similar across groups (exposed: 14 girls; unexposed: 9 girls), as were maternal minority background (both groups: *n* = 8) and maternal learning disability (exposed: *n* = 4; unexposed: *n* = 3). Infants’ birth order was comparable (exposed: *M* = 1.63, *SD* = 0.85; unexposed: *M* = 1.50, *SD* = 0.77).

Mothers with severe mental illness were slightly younger at childbirth (exposed: *M* = 30.65 years, *SD* = 4.97; unexposed: *M* = 32.99, *SD* = 4.05), and families showed somewhat lower socioeconomic status (exposed: median = 4.00, interquartile range = 2.37; unexposed: median = 5.00, interquartile range = 1.50). Infants in the exposed group were born slightly earlier (38.62 vs. 39.65 weeks) and had lower birthweight on average (2889 g vs. 3263 g). Paternal mental illness was more frequently reported in the exposed group (*n* = 9) than in unexposed controls (*n* = 2).

### Experimental paradigms

Experiments 1 and 2 were administered in a single session in counterbalanced order, with all participants included in data analysis completing both experiments. Despite attrition, counterbalancing was largely maintained: 16 exposed infants and 15 controls completed Experiment 1 first.

#### Experiment 1

In the first experiment, the differential response to voice vs. non-voice stimuli across exposed and unexposed infants was tested. Voice stimuli were adapted from the neutral condition in Maurage et al.^23^ and consisted in four consecutive presentations of the sound /a/ (duration: 2 s) with mean f0 at 193 Hz and mean formants at 801 Hz, 1427 Hz, 2877 Hz, and 4203 Hz. Non-voice stimuli were based on Blasi et al.^22^ and consisted in a sequence of environmental noises with matched duration and intensity to the ones of voice stimuli, and mean formants at 751 Hz, 1941 Hz, 2961 Hz, and 3880 Hz. Stimulus duration was 8 seconds for both conditions, followed by a silent baseline with duration between 9 and 11 s, to jitter the start of the upcoming stimulus. The paradigm included 8 trials per condition, and lasted approximately 6 minutes. By presenting voice stimuli with neutral semantic or prosodic valence and acoustically matched non-voice stimuli, we aimed at creating a sequence of stimuli in which neutral voice stimuli represented the social-communicatively salient condition, rather than a control condition to emotional stimuli, which could cause atypical response^30^.

#### Experiment 2

In the second experiment, stimuli were spoken realisations of a sentence with neutral semantic content and varying prosodic content. A female voice pronounced the sentence “Dogs are sitting by the door” with angry, happy, or neutral intonation (see Livingstone & Russo^24^, for further description and validation details). Stimulus duration was 10 s, followed by a silent baseline with a jittered duration ranging between 9 and 11 s. Each condition included 8 trials, and the experiment lasted approximately 9 minutes.

For both experiments, stimuli were presented at normalised intensity (70 dB), and conditions were presented in a pseudo-random order, with the constraint of not having the same condition more than 2 times in a row. The paradigms were presented while a silent movie (Inscapes)^31^ was displayed to help infants sit still.

### Data collection and processing

fNIRS data were acquired at 10.17 Hz sampling frequency on a NIRSport system (NIRx, Berlin) with a probe consisting of 8 sources and 8 detectors, which formed a total of 20 channels covering temporal lobes bilaterally, which are primarily involved in sound, language, prosody and emotional processing across development^27,32^. Optodes were mounted in a pre-perforated cap provided by the manufacturer, with their placement reflecting standardised scalp locations in the international 10-10 system (see Supplementary material S1). Signal processing was run in Homer2 toolbox for Matlab. To allow for a cluster-based approach for statistical analysis (Supplementary material S1), exclusion criteria were applied at the participant level rather than at channel level: children with signal-to-noise ratio (SNR) < 2 in any of the channels were excluded from analysis, and all children in the final sample contributed data from all channels. Out of the 7 exposed and 8 unexposed infants excluded due to poor data quality, 2 exposed and 3 unexposed infants were excluded because of SNR < 2.

We applied the processing chain described in Di Lorenzo et al.^33^. As a first step, intensity measurements at 760 nm and 850 nm wavelengths were converted to optical density (OD), with data processing being run namely on OD. Spline interpolation (parameter p = 0.99) was applied for drift correction and wavelet decomposition (with interquartile range = 0.5) was used for the correction of jump and remaining motions artefacts. After the corrections, the remaining steep signal changes (exceeding 5 *SD* per 0.5 s, or 0.30 OD units) were identified as artefacts, and trials including artefacts from 2 s before stimulus onset until 2 s after offset were discarded. Children with less than 3 valid trials per condition after artefact rejection were excluded from analysis; this was the case for 5 exposed and 5 unexposed children (out of 7 and 8 excluded in each group during fNIRS data processing). OD data were band-pass filtered (0.01–0.5 Hz) for the exclusion of physiological artefacts, converted to concentration data (preferential pathway factors: 5.3 for HbO, 4.2 for Hbb)^34^ and averaged by condition for each participant. Individual data contributed to the grand-average for group-level analysis. All processing steps were applied using Homer2 functions.

The number of valid trials ranged between 3 and 8 for all conditions and groups. In Experiment 1, the mean number of trials for exposed infants was 7.10 (*SD* = 1.56) in the voice and 7.06 (*SD* = 1.57) in the non-voice conditions, and for unexposed infants the mean was 6.83 (*SD* = 1.72) voice and 6.60 (*SD* = 1.85) non-voice trials. In Experiment 2, exposed infants had on average 6.06 (*SD* = 1.96) valid neutral, 5.66 (*SD* = 1.97) happy, and 5.70 (*SD* = 1.98) angry trials; unexposed infants had 6.03 (*SD* = 2.04) neutral, 6.20 (*SD* = 2.04) happy, and 6.10 (*SD* = 1.97) angry trials.

### Statistical analysis

Processed signal was averaged over a time-window of interest which started 2 s after stimulus onset, to account for the start of the haemodynamic response, and lasted for the duration of the stimulus. This corresponds to 2–10 s after stimulus onset for the voice/non-voice paradigm and to 2–12 s for the emotional voice paradigm, respectively.

For each paradigm, as a first step, mixed-model ANOVA was run on HbO and Hbb concentration over the entire probe to test for main effects of stimulus, group, and a stimulus-by-group interaction, using the non-parametric, assumption free cluster-based permutation approach in Fieldtrip toolbox for MATLAB to correct for multiple comparisons^35^. This allowed for a preliminary, data-driven identification of clusters of channels of interest. Secondarily, it was tested whether the stimulus-by-group interaction remained significant when controlling for variables that are predictive of language development: socio-economic status, maternal learning disability, minority background, paternal mental illness, infant sex, and birthweight. Data on gestational age at birth were also collected, but, due to its correlation with birthweight (*r*(60) = 0.599, *p* < 0.001) and concerns on data quality (i.e. uncertainty about whether all mothers had undergone first trimester ultrasound dating), only birthweight was retained, as it reflects both gestational age and possible foetal growth conditions, while being more reliably measured than gestational age.

In addition to the main effects of each factor and covariate, all the two-way interactions between stimulus condition and each of the variables of interest were included as planned comparisons.

Statistical tests based on mean distribution, rather than rank-based non-parametric tests were used throughout (i.e., independent-samples *t* tests and Pearson’s *r*) to maximise statistical sensitivity and maintain comparability across analyses. To ensure robust inference, statistical significance was evaluated using cluster-based permutation (for initial tests on fNIRS data) or bootstrapped confidence intervals (1000 resamples), which rely on minimal distributional assumptions and provide an empirical estimate of the sampling distribution of the statistics.

## Results

The final sample included 30 exposed and 30 unexposed mother–infant pairs (Table 1). Included infants contributed data to both fNIRS experiments. Mothers with severe mental illness were younger and had lower indicators of socio-economic status. At birth, exposed infants showed lower gestational age and birthweight. At 12 months, follow-up data were available for 47 infants (21 exposed, 26 unexposed). Exposed infants scored lower on verbal IQ, with a mean of 83.42 (*SD* = 13.72) versus 89.73 (*SD* = 11.88) in unexposed infants, but the difference was not significant (*t*(45) = − 1.65, *p* = 0.099, *α* = 0.05, *d* = −0.49, 95% CI [ − 1.07, 0.09]; *BF₀₁* = 1.09, Cauchy prior *r* = 0.707). Non-verbal IQ did not differ between groups (*t*(45) = 0.233, *p* = 0.817, *α* = 0.05, *d* = 0.06, 95% CI [–0.50, 0.64]; *BF₀₁* = 3.36, Cauchy prior, *r* = 0.707), with a mean of 98.09 (*SD* = 9.78) in exposed and 97.23 (*SD* = 14.50) in unexposed infants.

### Experiment 1

**Fig. 1:**
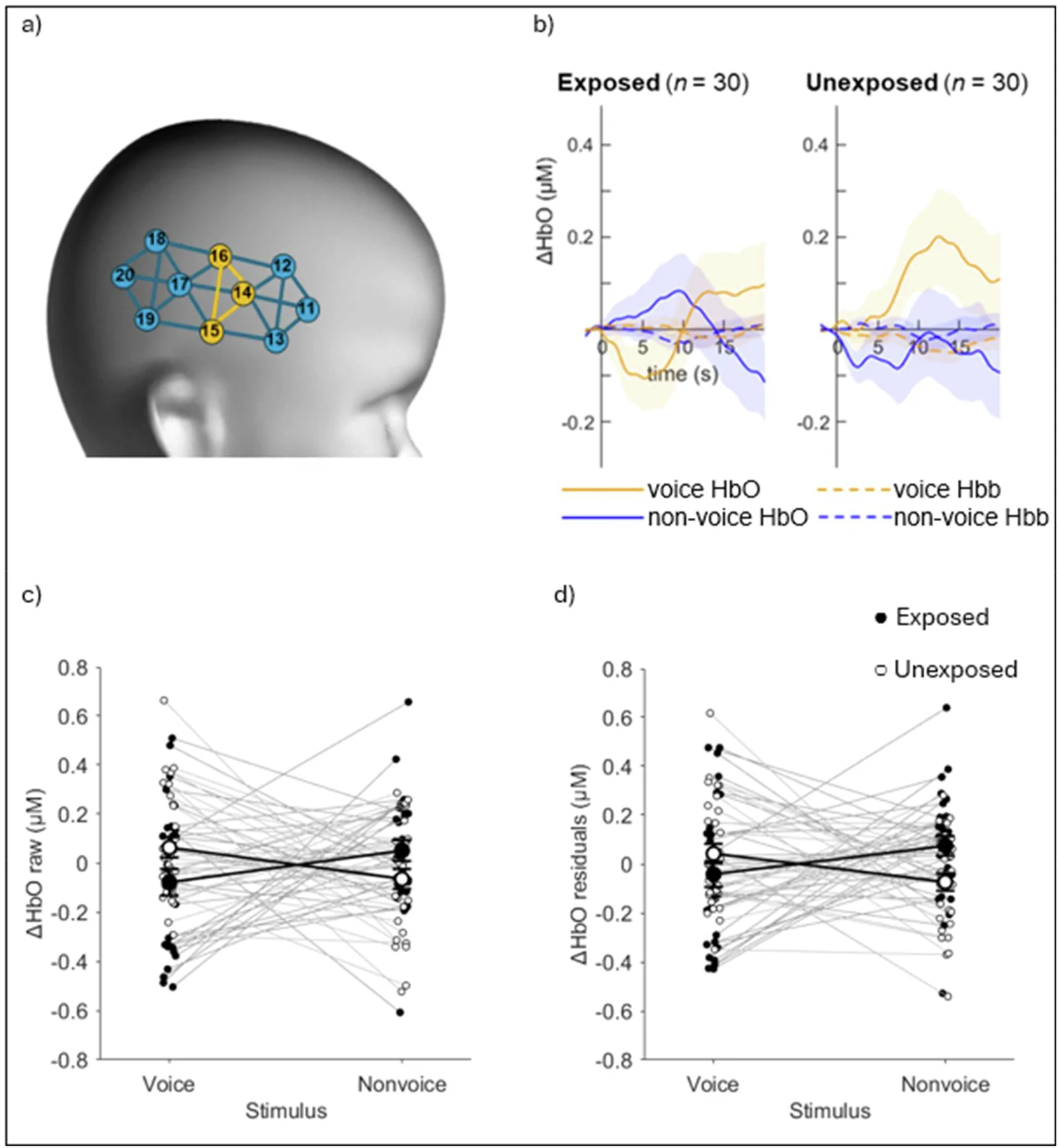
Response to voice and non-voice sounds in exposed (*n* = 30) and unexposed infants (*n* = 30). Position of channels 14, 15, and 16 in the probe (**a**):they formed a cluster of adjacent channels. Mean response to voice and non-voice sounds (lines) and *SEM* (shadowed areas) in the two groups (**b**): unexposed infants show greater activation to voice than to non-voice, while differential activation is not observed in exposed infants. Mean and *SEM* for response to voice and non-voice in exposed and unexposed infants (**c**), who display an opposite relation between the responses to the two stimulus types. Mean and *SEM* of residuals for response to voice and non-voice in exposed and unexposed infants (**d**) after controlling for infant sex, birthweight, socioeconomic status, maternal minority background, learning disability, and paternal mental illness. Of the two stimulus types, group difference is more prominent for non-voice stimuli.

There was evidence that the voice and non-voice stimuli used in the Experiment 1 paradigm elicited different HbO responses in exposed and unexposed children, whereas no significant main effect or interaction of stimulus and group was identified for Hbb values. Specifically, a significant stimulus-by-group interaction was observed for HbO concentration over a cluster encompassing channels 14, 15, and 16 (Fig. 1a), located in the medial and posterior right temporal areas (cluster’s summed *F*(1,58) = 11.24, *p* = 0.039, *α* = 0.05, *η*^*2*^_*p*_ = 0.11, 95% CI [0.03, 0.24]).

The averaged HbO signal over the three channels in the cluster (Fig. 1b) was used for further analysis, which showed that the stimulus-by-group interaction remained significant after controlling for socioeconomic status, birthweight, infant’s sex, paternal mental illness, maternal learning disability and minority background (*F*(1,52) = 7.47, *p* = 0.009, *α* = 0.05, *η*^*2*^_*p*_ = 0.12, 95% CI [0.02, 0.25]). There was also a significant stimulus-by-socioeconomic status interaction (*F*(1,52) = 4.04, *p* = 0.049, *α* = 0.05, *η*^*2*^_*p*_ = 0.07, 95% CI [0.001, 0.19]).

The effect of stimulus, after controlling for infant’s sex, birthweight, socioeconomic status, maternal learning disability, minority background, and paternal mental illness, was significant within the unexposed group (*F*(1,23) = 7.24, *p* = 0.013, *α* = 0.025, *η*^*2*^_*p *_= 0. 24, 95% CI [0.05, 0.47]), who showed greater activation in response to voice (residualised HbO values for voice: *M* = 0.04, *SD* = 0.28; non-voice: *M* = –0.07, *SD* = 0.19; raw HbO for voice: *M* = 0.06, *SD* = 0.22; non-voice: *M* = –0.06, *SD* = 0.22), but not within the exposed group (*F*(1,23) = 0.06, *p* = 0.800, *α* = 0.025, *η*^*2*^_*p*_ = 0.003; residualised HbO values for voice: *M* = –0.04, *SD* = 0.22; non-voice: *M* = 0.07, *SD* = 0.19; raw HbO for voice: *M* = –0.07, *SD* = 0.29; non-voice: *M* = 0.05, *SD* = 0.23). A one-sided Bayesian paired-sample *t* test using a default Cauchy prior (*r* = 0.707) on residualised HbO concentration values in response to voice and non-voice provided support for the lack of preference for voice stimuli in exposed infants (*BF*_*01*_ = 12.42).

Univariate ANCOVAs for voice and non-voice, with all variables of interest accounted for, showed a significant effect of group in the response to non-voice stimuli (*F*(1,52) = 8.95, *p* = 0.004, *α* = 0.025, *η*^*2*^_*p*_ = 0.15, 95% CI [0.03, 0.29]), but not to voice stimuli (*F*(1,52) = 1.88, *p* = 0.176, *α* = 0.025, *η*^*2*^_*p*_ = 0.035, 95% CI [0, 0.14]). Figure 1c, d displays the mean HbO concentration values for voice and non-voice stimuli in the two groups, based on raw data and on residuals after controlling for other variables of interest, respectively.

To further assess the contribution of maternal mental health to responses to voice and non-voice stimuli, HADS subscale scores were entered separately in place of maternal diagnostic status. The stimulus × group interaction remained significant for anxiety scores (*F*(1,52) = 7.06, *p* = 0.010, *α* = 0.025, *η*^*2*^_*p*_ = 0.12, 95% CI [0.02, 0.25]), which, with the other variables of interest partialled out, were associated with reduced responses to voice stimuli (*r*(60) = –0.315, *p* = 0.014, *α* = 0.05, 95% bootstrapped CI: –0.543, –0.044), rather than peculiarities in non-voice stimulus processing, suggesting that the stimulus  ×group interaction in the main analysis reflects a more complex combination of traits, beyond anxiety alone, in mothers with severe mental illness.

For non-voice stimuli, there was also a significant effect of socio-economic status (*F*(1,52) = 8.28, *p* = 0.006, *α* = 0.025, *η*^*2*^_*p*_ = 0.13, 95% CI [0.03, 0.27]), which may underlie the stimulus × socio-economic status interaction observed in the ANOVA. The effect of socio-economic status was non-significant for voice stimuli (*F*(1,52) = 0.21, *p* = 0.645, *α* = 0.025, *η*^*2*^_*p*_ = 0.004, 95% CI [0, 0.07]). To further expore the direction of the effect of socio-economic status on the response to non-voice stimuli, partial correlation analysis was run between the two variables, with maternal diagnostic status, learning disability, minority background, paternal mental illness, infant’s sex and birthweight controlled for. This showed positive correlation between socio-economic status and response to non-voice (*r*(60) = 0.371, *p* = 0.006, *α* = 0.025, 95% bootstrapped CI [0.153, 0.590]).

Post-hoc, the correlation between socioeconomic status and non-voice stimuli was further tested within each group, which showed entirely positive 95% CI for the correlation coefficient both for unexposed (*r*(30) = 0.562, *p* = 0.003, *α* = 0.025, 95% bootstrapped CI [0.022, 0.794]) and for exposed infants (*r*(30) = 0.300, *p* = 0.145, *α* = 0.025, 95% bootstrapped CI [0.015, 0.651]), which supports the robustness of the effect.

The relation between stimulus processing at 9 months and language development at 12 months was explored for all channels in the probe for the 47 participants with longitudinal data: the cluster of channels with an effect of maternal mental illness did not show any correlation with non-verbal IQ or verbal IQ, but channel 13, adjacent to the cluster, displayed a significant correlation between response to non-voice sounds and verbal IQ (*r*(47) = 0.576, *p *< 0.001 *α* = 0.0025, 95% bootstrapped CI [0.286, 0.778]), also after partialling out maternal diagnostic status, socio-economic status, minority background, learning disability, paternal mental illness, infant’s sex and birthweight (*r*(47) = 0.598, *p *< 0.001 *α* = 0.0025, CI = [0.281, 0.754]). Importantly, the correlation remained significant when it was tested within each group separately, with relevant variables partialled out (exposed: *r*(21) = 0.537, *p* = 0.039, FDR-corrected *α* = 0.05, 95% bootstrapped CI [–0.099, 0.925]; unexposed: *r*(26) = 0.710, *p* < 0.001, FDR-corrected *α* = 0.05, 95% bootstrapped CI [0.109, 0.887]). While both verbal IQ and socioeconomic status correlated with response to non-voice stimuli, albeit measured at slightly different locations, they were not mutually correlated in this sample (*r*(47) = 0.146, *p* = 0.327, *α* = .05, 95% bootstrapped CI [–0.094, 0.430]).

Supplementary material S2 shows HbO and Hbb responses for channels and stimulus conditions in exposed and unexposed infants.

### Experiment 2

In Experiment 2, stimulus condition (neutral, angry, happy prosody) was included as a within-subject factor. As the assumption of sphericity was violated (Mauchly’s *W* = 0.836, *p* = 0.010), the Greenhouse–Geisser correction (*ε* = 0.859) was applied in all repeated-measures ANOVA and ANCOVA analyses.

**Fig. 2:**
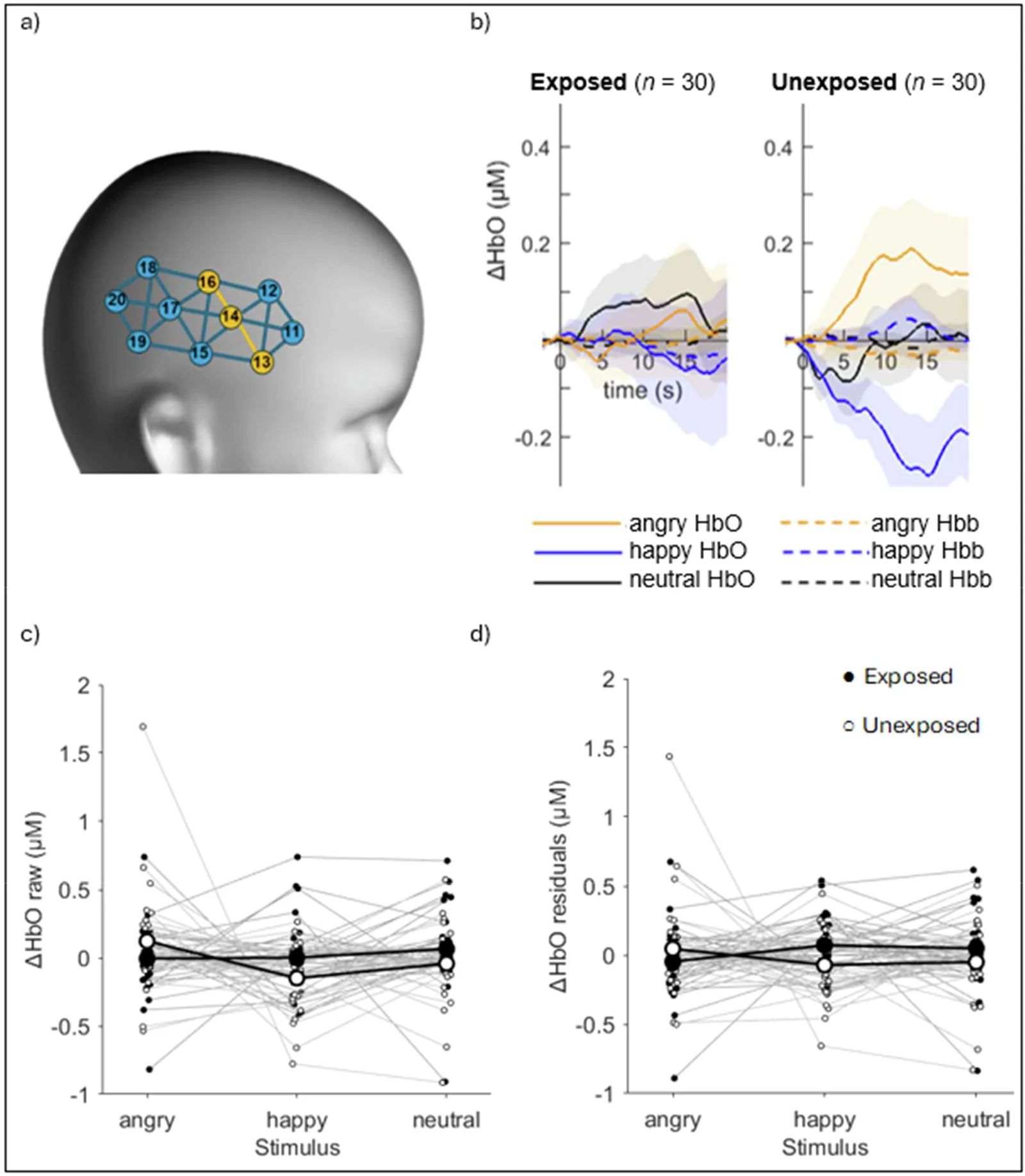
Response to angry, happy, and neutral voices in exposed (*n* = 30) and unexposed infants (*n* = 30)*.* Position of channels 13, 14, and 16 in the probe and their organisation into a cluster (**a**). Mean response to the spoken sentence “Dogs are sitting by the door” pronounced with angry, happy, and neutral prosody in exposed and unexposed infants (**b**). Shadowed areas correspond to SEM. Means, *SEM*, and individual data for raw HbO concentration in the three stimulus conditions in the two groups (**c**). Means, *SEM*, and individual data for residualised HbO concentration across stimulus conditions and groups (**d**).

A cluster of channels (n, 13, 14, and 16) over the right temporal lobe showed a stimulus × group interaction in relation to HbO concentration which was significant in the unadjusted model (*F*(1.77,101.40) = 12.14, *p* = 0.048, *α* = 0.05, *η*^*2*^_*p*_ = 0.053, 95% *CI* [0.001, 0.118]). The cluster (Fig. 2a) is topographically consistent with the one showing voice × group interaction in Experiment 1. No significant effects or interactions were observed for Hbb concentration. Figure 2b displays HbO and Hbb responses across stimulus conditions, averaged over the channels in the significant cluster. When controlling for socioeconomic status, minority background, maternal learning disability, infant’s sex, birthweight, and paternal mental illness, the stimulus × group interaction for HbO responses shifted slightly above *α* (*F*(1.72,89.34) = 3.11, *p* = 0.057, *α* = 0.05, *η*^*2*^_*p*_ = 0.057, 95% CI [0, 0.13]), while a significant main effect of socioeconomic status emerged (*F*(1,52) = 5.38, *p* = 0.024, *α* = 0.05, *η*^*2*^_*p*_ = 0.095, 95% CI [0.01, 0.23]).

Simple contrast (angry minus neutral and happy minus neutral) for the stimulus × group interaction in the uncorrected ANOVA (Fig. 2c), with group as a categorical moderator, suggests that the stimulus × group interaction might be driven by the different relation between angry and neutral stimulus response across exposed and unexposed infants (*t*(116) = –2.37, *p* = 0.019, *α* = 0.025, *β* = –0.279, 95% CI [–0.047, –0.512]). The relation between response to happy and neutral stimuli did not significantly differ across groups (*t*(116) = 0.37, *p* = 0.708, *α* = 0.025, *β* = 0.04, 95% bootstrapped CI [–0.18, 0.27]). Mean raw HbO concentration (Fig. 2c) in the exposed group was 0.06 μM (*SD* = 0.28) for neutral, 0.002 μM (*SD* = 0.26) for happy and –0.006 μM (*SD* = 0.25) for angry stimuli. In the unexposed group, mean raw HbO concentration was –0.04 μM (*SD* = 0.28) for neutral, –0.14 μM (*SD* = 0.23) for happy, and 0.12 μM (*SD* = 0.39) for angry stimuli. Residualised HbO concentration (Fig. 2d) in the exposed group was 0.05 μM (*SD* = 0.27) for neutral, 0.07 μM (*SD* = 0.19) for happy, and –0.04 μM (*SD* = 0.26) for angry stimuli, while in the unexposed group the residual values were –0.05 μM (*SD* = 0.27), –0.07 μM (*SD* =  0.23), and 0.04 μM (*SD* = 0.35), respectively. Supplementary material S3 displays HbO and Hbb time courses over the entire fNIRS probe in both groups of infants.

**Fig. 3:**
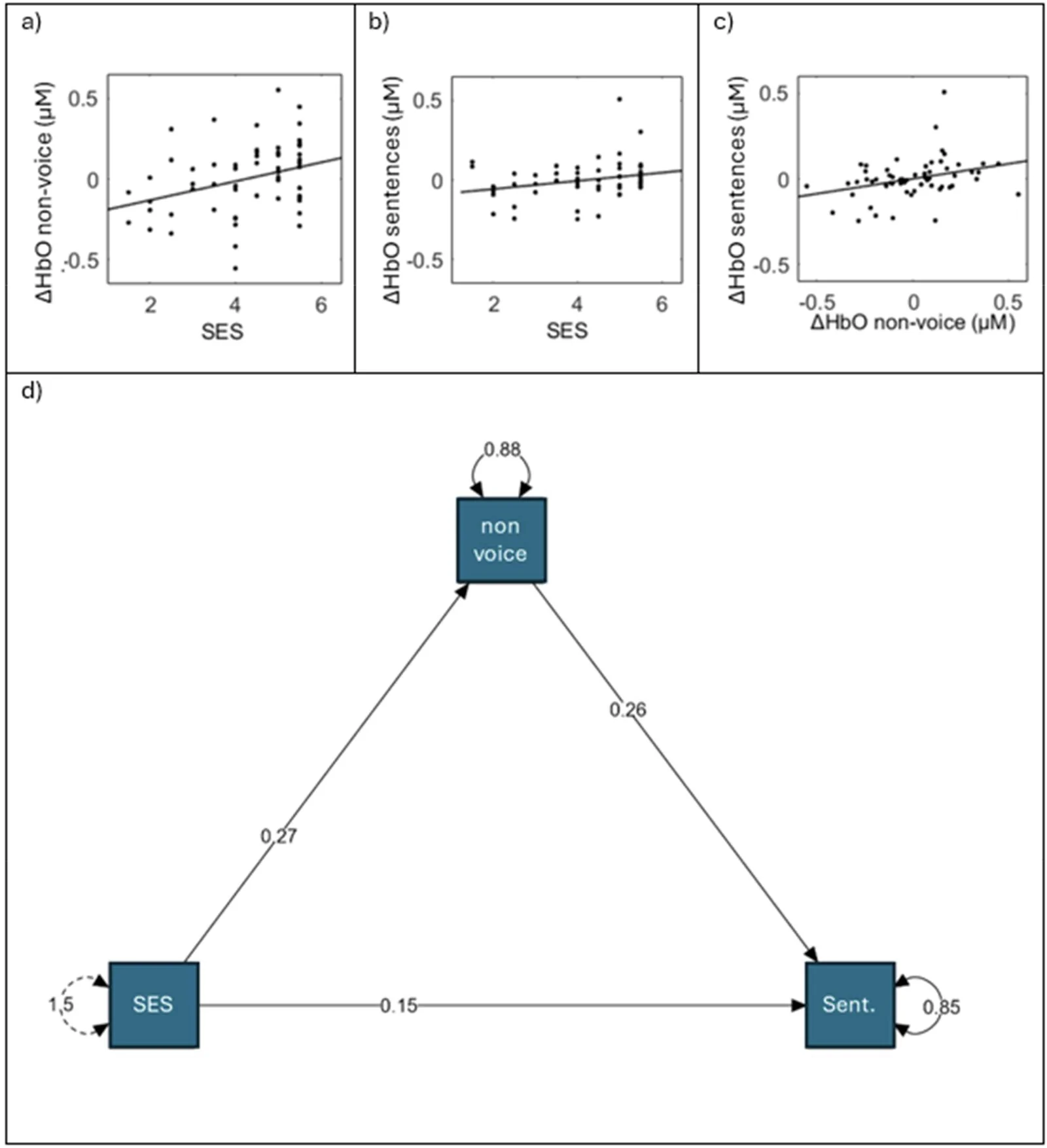
The relation between maternal diagnostic status (group), response to non-voice stimuli, mean response to sentences, and socioeconomic status (SES) across the entire sample (exposed: *n* = 30, unexposed: *n* = 30). Partial correlation between socioeconomic status (SES) and response to non-voice, with group and all other relevant variables controlled for (**a**). Partial correlation between SES and the averaged response across linguistic stimuli, which is, sentence stimuli with happy, angry, and neutral prosody in the emotional intonation paradigm (**b**). Interrelation between response to non-voice and the averaged response to sentences, with group and all other variables of interest partialled out (**c**). Mediation of response to non-voice in the correlation between SES and mean response to sentences (**d**).

As a follow-up for the main effect of socio-economic status (Fig. 3a, b and c), partial correlation analysis was run between socio-economic status and the averaged signal across conditions, while controlling for maternal diagnostic status, learning disability, minority background, paternal mental illness, infant sex and birthweight. This indicated a positive relation between socio-economic status and mean stimulus response (*r*(60) = 0.309, *p* = 0.023, *α* = 0.05, CI [0.003, 0.511]). Exploratory analysis showed no correlation between activation in response to emotional prosody and verbal IQ.

Because the response to linguistic stimuli averaged across conditions in experiment 2 and response to non-voice stimuli in experiment 1 correlated with socio-economic status, their mutual correlation was tested post hoc. The correlation was significant (*r*(60) = 0.35, *p* = 0.006, *α* = 0.05, 95% CI [0.16, 0.51]). Mediation analysis was also run, to test whether response to non-voice mediated the relation between socio-economic status and the averaged response to linguistic stimuli. To do so, the other variables of interest (maternal diagnostic status, minority background, learning disability, infant’s sex, birthweight, and paternal mental illness) were first regressed out, and residual values were used. To evaluate mediation effects, we focused on the distribution of bootstrapped CIs over *p *values. This choice is motivated by the fact that *p *values rely on normality assumptions, whereas the sampling distribution of indirect effects is typically non-normal, making bootstrap-based inference more appropriate^36^. Accordingly, evidence for mediation could be inferred if the indirect effect of socio-economic status (via responses to non-voice stimuli in Experiment 1) on the averaged response to linguistic stimuli across conditions was entirely positive or entirely negative—consistent with the total effect (i.e., without accounting for the mediator)—while the direct effect of socio-economic status (i.e., controlling for responses to non-voice stimuli in Experiment 1) included 0. This pattern was observed. The 95% bootstrapped CI of the regression coefficient was entirely positive for both the total effect (*β* = 0.22, *p* = 0.034, *α *= 0.05, 95% CI [0.04, 0.45]) and the indirect effect (*β* = 0.07, *p* = 0.101, *α *= 0.05, 95% CI [0.01, 0.18]), whereas it included 0 for the direct effect (*β* = 0.15, *p* = 0.165, *α* = 0.05, 95% CI [–0.03, 0.37]), supporting mediation (Fig. 3d).

## Discussion

This study provides evidence of atypical neural processing of voice in infants of mothers with severe mental illness, controlling for additional factors that may influence language development or the likelihood for neurodevelopmental disorders, namely infant sex, birthweight, socio-economic status, minority background, maternal learning disability and paternal mental illness. In this approach, infants born to mothers with severe mental illness are considered as a distinct group with elevated likelihood for atypical neurodevelopment, not merely explained by sociodemographic factors.

At 9 months, infants exposed to severe maternal mental illness showed no preferential response to voice stimuli, unlike controls. After adjusting for additional factors linked to neurodevelopmental disorder or language delay, the group difference in response to voice was attenuated, but exposed infants showed an atypically enhanced response to non-voice stimuli. Similar patterns—lack of clear preference for social stimuli, but increased non-social reactivity—have been reported in other elevated-likelihood groups, such as siblings of autistic children^37^. Given the effect sizes observed here and in related studies^9,37^, the incidence of neurodevelopmental disorders in infants exposed to severe maternal illness may be comparable to the one observed in siblings of autistic children, which is close to 20% for autism spectrum disorder^3^ and to 10% for developmental delay^38^, although this remains to be verified longitudinally.

While it is known from larger studies that language atypicalities in children of mothers with severe mental illness become observable during the second year of life^39^, verbal IQ data collected at 12 months in the current sample suggest that exposed infants may show signs of language delay, but do not provide conclusive evidence on this. Moreover, the observed neural differences between exposed and unexposed infants at 9 months do not seem related to verbal IQ at 12 months. Yet, neural measures at a right antero-temporal location (channel 13), adjacent to the cluster at which the groups differed were robustly correlated with language development, also after controlling for potential confounds and testing the relation within each group separately, suggesting that activation in response to voice versus non-voice in this area may serve as a candidate early marker of developmental outcome. In addition to an interaction with group, stimulus type showed an interaction with socio-economic status, which was positively associated with neural response to non-voice stimuli, both across the sample and within groups.

There was a main effect of socioeconomic status on HbO concentration elicited by spoken sentences in experiment 2, independently of prosody, in a cluster largely overlapping with the one showing differential activation to voice and non-voice stimuli across groups. The cluster was initially identified because it presented a significant stimulus by group interaction, which however was no longer significant after controlling for potential confounds. Lower socio-economic status was associated with reduced responses to all stimuli, and mediation analyses indicated that it influenced speech processing indirectly through responses to non-voice sounds. Infants born to mothers with higher socio-economic status showed stronger non-voice activation, which in turn facilitated activation to spoken sentences—though not necessarily emotion differentiation. The role of non-social stimulus processing in paradigms focused on social perception has repeatedly emerged in developmental neuroscience. For example, Blasi et al.^40^ found that infants with elevated likelihood for neurodevelopmental disorder who showed stronger activation to non-voice sounds demonstrated higher social engagement during mother-child interactions. Similarly, Jones et al.^41^ observed that autistic toddlers with heightened sensory sensitivity later exhibited more typical patterns of social attention as preschoolers. In line with these findings, our results on the relation between socio-economic status, response to non-voice sounds, and processing of spoken sentences, suggest that enhanced response to non-social stimuli may play a protective role when social stimulus processing is atypical.

The observed right-lateralised pattern aligns with evidence implicating the right temporal lobe in prosodic and emotional processing^42,43^ and with atypical right-hemisphere activity in adults with mood or psychotic disorders^44–46^. This may indicate developmental continuity between early neural atypicalities in infants exposed to severe maternal mental illness and neural signatures of psychiatric conditions, consistent with shared risk factors across neurodevelopmental disorders and mental illness^47,48^.

### Limitations

While the results discussed here provide an initial description of the peculiarities of language processing in infants exposed to severe maternal mental illness, a key limitation of this study is the absence of long-term developmental data, as follow-up was disrupted by the COVID-19 pandemic. Although language delay in the exposed sample was not clearly observed at 12 months, larger studies have reported clearer effects of maternal mental illness on language development emerging from 18 months of age.

Importantly, while socio-economic status affected stimulus processing in both groups, mothers with severe mental illness had on average lower academic qualifications and household income than controls, despite our efforts to recruit exposed and unexposed infants from similar socio-economic backgrounds. The link between mental illness and socio-economic indicators appeared particularly close, and it remains difficult to disentangle directionality—whether mental illness constrains an individual’s opportunities, or whether socio-economic adversity impacts mental health. It is likely that these factors reinforce each other recursively.

Moreover, the spatial constraints of fNIRS limit conclusions to cortical regions within the recorded areas. While sound and voice processing, including emotional prosody, primarily engages temporal regions, frontal and pre-frontal areas may also begin to show differential responses between exposed and unexposed infants in the second half of the first year of life. Including frontal and pre-frontal regions in future probes would be particularly desirable to address the impact of severe maternal mental illness on the development of fronto-temporal functional connectivity.

Finally, future studies should adopt a broader perspective by including both maternal and paternal mental health, alongside clearer characterisation of each parent’s involvement in child-rearing.

## Conclusion

Despite the need for longitudinal data, this study characterises infants exposed to severe maternal mental illness as a group with early identifiable neural atypicalities in voice and language processing. It also describes the role of concurrent risk factors in the early stages of the neurodevelopmental pathway in exposed infants. Ideally, these findings will contribute to a greater recognition of the challenges faced by this group.

## Supplementary information

Transparent Peer Review file

Supplementary Materials

## Data Availability

Data^49^ is available at https://doi.org/10.17632/zcct58pryk.1.

## Code availability

Code^49^ is available at https://doi.org/10.17632/zcct58pryk.1.
