# Supplementary material for: Early detection of distinct language development in infants of mothers with severe mental illness using fNIRS
Source: Commun Psychol. 2026 Jun 15;4:131. doi: 10.1038/s44271-026-00475-y (PMC13630572; doi:10.1038/s44271-026-00475-y)
Supplement: Supplementary file 1 [file 44271_2026_475_MOESM1_ESM.pdf]

nature portfolio

Peer Review File

Early detection of distinct language development in infants of mothers with severe mental illness using fNIRS.

Corresponding Author: Dr Alessandra Piatti

This file contains all editorial decision letters in order by version, followed by all author rebuttals in order by version.

Version 0:

Decision Letter:

** Please ensure you delete the link to your author homepage in this e-mail if you wish to forward it to your coauthors **

Dear Dr Piatti,

Thank you for your patience during the peer-review process. Your manuscript titled "Early detection of distinct language development in infants of mothers with severe mental illness using fNIRS." has now been seen by 3 reviewers, and I include their comments at the end of this message. They find your work of interest but raised some important points. We are interested in the possibility of publishing your study in Communications Psychology, but would like to consider your responses to these concerns and assess a revised manuscript before we make a final decision on publication.

We therefore invite you to revise and resubmit your manuscript, along with a point-by-point response to the reviewers. Please highlight all changes in the manuscript text file.

The key issues requiring revision are clearer methodological justification and reporting, particularly regarding the fNIRS procedures, paternal mental-illness measures, and overall study design. In addition, the manuscript should present a more conservative interpretation of the findings and a fuller, literature-integrated discussion.

As you revise the manuscript in response to these issues, please also implement all requests in the attached Mandatory Revision Requests document. All requirements listed in this document need to be fully met, or the work will be returned to you for further revisions without peer review. This workflow is in place to increase the likelihood that the paper will be accepted for publication. It reduces the number of rounds of revision (and review) and ensures that the reviewers vet a version of the article that is compliant with journal policies. If you have any questions regarding the required revisions, please contact the journal prior to resubmission to avoid a negative outcome.

We are committed to providing a fair and constructive peer-review process. Please don't hesitate to contact us if you wish to discuss the revision in more detail.

Please submit the following items:

- Revised manuscript
- Point-by-point response to the referees' comments
- Mandatory Revision Requests Table (attached).
- Cover letter (as a separate document)

via this link: Link Redacted .

** This url links to your confidential home page and associated information about manuscripts you may have submitted or are reviewing for us. If you wish to forward this email to co-authors, please delete the link to your homepage first **

We hope to receive your revised paper within 8 weeks; please let us know if you aren't able to submit it within this time so that we can discuss how best to proceed. If we don't hear from you, and the revision process takes significantly longer, we may close your file. In this event, we will still be happy to reconsider your paper at a later date, provided it still presents a significant contribution to the literature at that stage.

We look forward to seeing the revised manuscript and thank you for the opportunity to review your work.

Best regards,

Shir Atzil

Shir Atzil
External Editor
Communications Psychology

REVIEWER EXPERTISE:
Reviewer #1: Developmental social neuroscience
Reviewer #2: Developmental cognitive neuroscience
Reviewer #3: fNIRS

REVIEWER REPORTS:

Reviewer #1 (Remarks to the Author):

The study addresses a very interesting and important topic, using both behavioral and neuroimaging tools to investigate early responses to speech in population of infants of mothers with mental illness compared with controls. While the topic is important, and the findings show important differences in voice processing between the two groups, I have some concerns, mostly related to missing information on the participating families.
Importantly, the authors should add information on mothers' medication treatment (both groups), including medication during pregnancy, and explore whether it influences the observed effects.
Additionally, it is important to include information about marital status and siblings. There is growing evidence for the father's important role in child development, including as a protective factor for maternal mental health. Therefore, it would be valuable to know whether the father is involved in child-rearing and, if possible, to obtain their general anxiety scores as well.
Another important point, since SES is a major component in this study and its findings, it is important to describe in the methods how the construct was calculated. Similarly add details on how each of the other variables were calculated- for example, how the score for Paternal mental illness was calculated?
In the Participants section, information is missing regarding the infants' gender, mean age and age range, as well as the mothers' age, marital status, and SES mean and range, reported separately for each group. Some of this information first appears in Table 1 in the Results section, but it should be included in the Participants description, with the range provided for each variable.
Some general information about the study procedure is missing. For example, whether both experiments were conducted on the same day within a single session or on separate days, and whether the same participants completed both experiments.
The abstract needs some edits for clarification. For example, what does 'enhanced processing of linguistic stimuli' mean, does it refer to the contrast between voice and non-voice stimuli, or to sentences with different emotional prosody? Similarly, the terminology in the abstract and introduction is confusing: sensitivity, discrimination, and processing seem to be used interchangeably. Are they all meant to refer to the same construct?
Adding more information to the y-axis in Figures 1c and 1d would be valuable, as it would make it easier to see the differences between them at a glance.

Reviewer #2 (Remarks to the Author):

In this study, fNIRs is used in two experiments to examine the effects of maternal severe mental illness (SMI) on infants' (aged 8-10 months) neural responses to voice versus non-voice sounds and sentence prosody. Infants exposed to SMI showed elevated responses to non-voice stimuli compared to controls. Furthermore, SMI-exposed infants demonstrated less clear differentiation between angry and neutral prosody in a largely overlapping sensor cluster. Clear relationships with language development at 12 months were not found, but SES was found to modulate neural responses. I thought this was an interesting paper on a very important topic, and it is clearly written. However, I would like to have more information concerning methods, some figure changes, and additional discussion of results before deciding if it is suitable for publication.

Methods:
What were the criteria for discarding data (i.e. based on insufficient quality or quantity of data). As mixed models were used, were infants still included if they had data at least from some conditions, or was that infant removed entirely?
How was prosody type confirmed for stimuli in experiment 2?
Was experiment 1 always done first? If so, did infant fatigue/inattention influence results in experiment 2?
A silent movie was played during data acquisition, but what was this movie? If, for example, it involved social stimuli like faces, I think this would be a serious confounding factor.
The authors do not explain why the temporal load sensors only were selected. Please add information about this.
Why was gestational age not controlled for even though this was lower in the SMI-exposed group?
Why were 8-10 months and 12 months in particular selected for assessment? Was 12 months a good choice considering that this is still a very early stage in terms of language development?

Figures:
In figures 1 and 2, please plot the individual data points for each infant

Discussion:
The evidence for SMI-exposed infants showing signs of language delay seems very weak, especially when considering the effects of SES as well. How strongly then can this be interpreted to be specific to SMI rather than SES?
SMI mothers had lower SES- what are the authors thoughts about directionality of effects?
Being able to record from only a small area of the scalp limits the conclusions that can be made about these findings (e.g. were responses specific to temporal areas, and if so/if not, what would this suggest). Please add some discussion on this issue.

Reviewer #3 (Remarks to the Author):

1 Why did the authors focus on the bilateral temporal cortex and neglect the left prefrontal cortex, a region related with language closely?
2 The authors should claim the reference points of the arrangement of fNIRS optode patches.
3 To enhance readability, the authors could provide more details about the logic or reason supporting the series of data processing. The authors stated that the processing pipeline was adapted from the previous study. In this case, they were suggested to claim the adopted and adapted contents. Also, were these preprocessing methods contradict to each other?
4 If possible, listing the valid fNIRS data for further analysis in each condition and experiment so that the readers could evaluate the soundness of the statistical analyses.
5 Please claim the reason why GLM was not used to portrait the brain activity?
6 Further analysis targeting functional connectivity is also recommended.

* TRANSPARENT PEER REVIEW: Communications Psychology uses a transparent peer review system. This means that we publish the editorial decision letters including Reviewers' comments to the authors and the author rebuttal letters online as a supplementary peer review file. However, on author request, confidential information and data can be removed from the published reviewer reports and rebuttal letters prior to publication. If your manuscript has been previously reviewed at another journal, those Reviewers' comments would not form part of the published peer review file.

** Visit Nature Research's author and referees' website at www.nature.com/authors for information about policies, services and author benefits**

Communications Psychology is committed to improving transparency in authorship. As part of our efforts in this direction, we are now requesting that all authors identified as ‘corresponding author’ create and link their Open Researcher and Contributor Identifier (ORCID) with their account on the Manuscript Tracking System prior to acceptance. ORCID helps the scientific community achieve unambiguous attribution of all scholarly contributions. You can create and link your ORCID from the home page of the Manuscript Tracking System by clicking on ‘Modify my Springer Nature account’ and following the instructions in the link below. Please also inform all co-authors that they can add their ORCIDs to their accounts and that they must do so prior to acceptance.
https://www.springernature.com/gp/researchers/orcid/orcid-for-nature-research

For more information please visit http://www.springernature.com/orcid

If you experience problems in linking your ORCID, please contact the Platform Support Helpdesk.

Version 1:

Decision Letter:

** Please ensure you delete the link to your author homepage in this e-mail if you wish to forward it to your coauthors **

Dear Dr Piatti,

Your manuscript titled "Early detection of distinct language development in infants of mothers with severe mental illness using fNIRS." has now been seen by our reviewers, whose comments appear below. In light of their advice I am delighted to say that we are happy, in principle, to publish a suitably revised version in Communications Psychology.

We therefore invite you to revise your paper one last time to address the remaining concerns of our reviewers and a list of editorial requests. At the same time we ask that you edit your manuscript to comply with our format requirements and to maximise the accessibility and therefore the impact of your work.

EDITORIAL REQUESTS:

Please review our specific editorial comments and requests regarding your manuscript in the attached "Editorial Requests Table". Please outline your response to each request in the right hand column. Please upload the completed table with your manuscript files as a Related Manuscript file.

If you have any questions or concerns about any of our requests, please do not hesitate to contact me.

SUBMISSION INFORMATION:

In order to accept your paper, we require the files listed here https://www.nature.com/documents/commsj-file-checklist.pdf .

OPEN ACCESS:

Communications Psychology is a fully open access journal. Articles are made freely accessible on publication. For further information about article processing charges, open access funding, and advice and support from Nature Research, please visit https://www.nature.com/commspsychol/open-access

At acceptance, you will be provided with instructions for completing the open access licence agreement on behalf of all authors. This grants us the necessary permissions to publish your paper. Additionally, you will be asked to declare that all required third party permissions have been obtained, and to provide billing information in order to pay the article-processing charge (APC).

* TRANSPARENT PEER REVIEW: Communications Psychology uses a transparent peer review system. On author request, confidential information and data can be removed from the published reviewer reports and rebuttal letters prior to publication. If you are concerned about the release of confidential data, please let us know specifically what information you would like to have removed. Please note that we cannot incorporate redactions for any other reasons.

* CODE AVAILABILITY: All Communications Psychology manuscripts must include a section titled "Code Availability" at the end of the methods section. We require that the custom analysis code supporting your conclusions is made available in a publicly accessible repository at this stage; please choose a repository that generates a digital object identifier (DOI) for the code; the link to the repository and the DOI must be included in the Code Availability statement. Publication as Supplementary Information will not suffice.

* DATA AVAILABILITY:
All Communications Psychology manuscripts must include a section titled "Data Availability" at the end of the Methods section. More information on this policy, is available in the Editorial Requests Table and at http://www.nature.com/authors/policies/data/data-availability-statements-data-citations.pdf.

Please use the following link to submit the above items:
Link Redacted
** This url links to your confidential home page and associated information about manuscripts you may have submitted or be reviewing for us. If you wish to forward this email to co-authors, please delete the link to your homepage first **

We hope to hear from you within two weeks; please let us know if you need more time.

Best regards,

Troby Lui, on behalf of

Shir Atzil

Troby Lui, PhD
Associate Editor
Communications Psychology

Shir Atzil
External Editor
Communications Psychology

REVIEWERS' COMMENTS:

Reviewer #1 (Remarks to the Author):

The authors have addressed most of my comments satisfactorily, and I have no further major concerns.

Reviewer #2 (Remarks to the Author):

I am very happy with the authors responses to my comments. The only change I would still like to see made before accepting for publication is moving the modified Figure 1 and 2 plots showing individual data points from the supplement to the main manuscript (to increase transparency).

Reviewer #3 (Remarks to the Author):

The author provided acceptable explanations and revisions for my comments, and I have no further suggestions.

** Visit Nature Research's author and referees' website at www.nature.com/authors for information about policies, services and author benefits**

Open Access This Peer Review File is licensed under a Creative Commons Attribution 4.0 International License, which permits use, sharing, adaptation, distribution and reproduction in any medium or format, as long as you give appropriate

credit to the original author(s) and the source, provide a link to the Creative Commons license, and indicate if changes were made.
In cases where reviewers are anonymous, credit should be given to 'Anonymous Referee' and the source.
The images or other third party material in this Peer Review File are included in the article's Creative Commons license, unless indicated otherwise in a credit line to the material. If material is not included in the article's Creative Commons license and your intended use is not permitted by statutory regulation or exceeds the permitted use, you will need to obtain permission directly from the copyright holder.
To view a copy of this license, visit https://creativecommons.org/licenses/by/4.0/

On behalf of all co-authors, I sincerely thank the Reviewers for their evaluation of our manuscript and for their constructive comments. We are grateful for the opportunity to revise our work.
In response to the Reviewers' comments, we have made extensive revisions across the manuscript. In particular, we have expanded the description of participant characteristics and key demographic variables, clarified methodological procedures and analytical choices, and improved the reporting of statistical analyses. We have also revised the abstract and main text for greater terminological consistency, refined the interpretation of results. We are also more explicitly addressing the limitations of this work. In addition, we have improved figure presentation and provided further details in the Supplementary Materials where appropriate. Below, we provide a detailed, point-by-point response to each comment. All changes in the manuscript are clearly indicated, and we hope that the revisions adequately address the reviewers' concerns.

Sincerely,

Alessandra Piatti

REVIEWER REPORTS:

Reviewer #1 (Remarks to the Author):

The study addresses a very interesting and important topic, using both behavioral and neuroimaging tools to investigate early responses to speech in population of infants of mothers with mental illness compared with controls.

1) **While the topic is important, and the findings show important differences in voice processing between the two groups, I have some concerns, mostly related to missing information on the participating families.**
   We are address the Reviewer's concerns below.

2) **Importantly, the authors should add information on mothers' medication treatment (both groups), including medication during pregnancy, and explore whether it influences the observed effects.**
   We agree that this is an important point and we are now referring to medication data in the Participants subsection. However, we feel that it would not be appropriate, for the design of our study, to further elaborate on the relation between maternal medication and language processing/ language development in infants. In fact, while the current sample size was sufficient to observe general, group effects, it is too limited to disentangle the role of each medication type. For instance, during explorative analyses, not included in the manuscript, we found a negative relationship between sertraline use in mothers and both fNIRS response to voice stimuli and verbal IQ at 12 months in infants. However, we do not feel that reporting these results would make an important contribution to the field, and may even generate unnecessary confusion among patients and families, particularly if such findings were to receive wider public attention.
   Importantly, our sample does not include mothers with a clinical diagnosis of depression who were not taking medication, which makes it difficult to tell apart the effects of diagnostic status and those of medication. Sertraline was also the most represented medication in the psychiatric sample, which likely explains why the association emerged specifically for this medication and not for other drugs, including serotonergic.
   Larger studies have found no association between sertraline use in mothers and language development in infants. We feel that raising concerns about drug safety based on a study not designed for this purpose would not be appropriate. Also, due exclusion criteria, no control mothers took psychiatric or neurologic medications: while medications for other conditions can also affect child development, our data are too sparse and likely biased in this regard (i.e.: 9 control mothers recalling paracetamol assumption and 2 recalling ondansetron assumption during pregnancy are not comparable with 30 mothers with mental illness whose medication list was available to use from medical records) to draw conclusions on developmental effects.

3) **Additionally, it is important to include information about marital status and siblings.**

   We have added information about birth order in Table 1. Nevertheless, as the Reviewer suggests, it would be necessary to integrate this information with more comprehensive data on child-to-caregiver ratio. While we did record data on marital status, that information is incomplete as it does not sufficiently account for resource

availability (e.g.: the father's actual involvement in child rearing can vary, but at the same time other adults than the parents can be available as support figures).

4) **There is growing evidence for the father's important role in child development, including as a protective factor for maternal mental health. Therefore, it would be valuable to know whether the father is involved in child-rearing and, if possible, to obtain their general anxiety scores as well.**

We agree that, although the present study primarily focuses on maternal mental health, paternal factors are also highly relevant for child development, including as potential protective influences.
Unfortunately, our data collection was centred on mothers, and we have limited information regarding fathers. While we did collect data on marital status, we do not have detailed measures of paternal involvement in child-rearing (e.g.: specific tasks, number of hours per day...). This was partly due to our decision to focus on the parent with a clear biological link to the child, without requiring additional genetic testing to confirm paternity, and who, in the societal context in which the study was run, is the primary user of parental leave and caregiver in the first months of the infant's life.
As a result, the only information available on fathers concerns the presence or absence of a psychiatric diagnosis, recorded in a dichotomous manner, and we do not have data on symptom severity (e.g., anxiety levels).
We have now made this limitation more explicit in the corresponding section of the manuscript.

5) **Another important point, since SES is a major component in this study and its findings, it is important to describe in the methods how the construct was calculated.**

In the revised manuscript, we provide a clearer description of these factors and covariates.

6) **Similarly add details on how each of the other variables were calculated- for example, how the score for Paternal mental illness was calculated?**

We have added this in the Methods section.

7) **In the Participants section, information is missing regarding the infants' gender, mean age and age range, as well as the mothers' age, marital status, and SES mean and range, reported separately for each group. Some of this information first appears in Table 1 in the Results section, but it should be included in the Participants description, with the range provided for each variable.**
We are now presenting Participants information in a separate table, to which we refer in the apposite section. The Results section is re-organised accordingly.

8) **Some general information about the study procedure is missing. For example, whether both experiments were conducted on the same day within a single session or on separate days, and whether the same participants completed both experiments.**
We have added this information in the Methods section.

9) **The abstract needs some edits for clarification. For example, what does 'enhanced processing of linguistic stimuli' mean, does it refer to the contrast between voice and non-voice stimuli, or to sentences with different emotional prosody? Similarly, the terminology in the abstract and introduction is confusing: sensitivity, discrimination, and processing seem to be used interchangeably. Are they all meant to refer to the same construct?**
   We have revised the abstract and the main text for terminological consistency.

10) **Adding more information to the y-axis in Figures 1c and 1d would be valuable, as it would make it easier to see the differences between them at a glance.**
    We have edited Figure 1c and 1d, specifying in the y-axis of Figure 1c that it refers to raw concentration values and in Figure 1d that it refers to residual values. Further information is available in the caption.

Reviewer #2 (Remarks to the Author):

In this study, fNIRs is used in two experiments to examine the effects of maternal severe mental illness (SMI) on infants' (aged 8-10 months) neural responses to voice versus non-voice sounds and sentence prosody. Infants exposed to SMI showed elevated responses to non-voice stimuli compared to controls. Furthermore, SMI-exposed infants demonstrated less clear differentiation between angry and neutral prosody in a largely overlapping sensor cluster. Clear relationships with language development at 12 months were not found, but SES was found to modulate neural responses. I thought this was an interesting paper on a very important topic, and it is clearly written. However, I would like to have more information concerning methods, some figure changes, and additional discussion of results before deciding if it is suitable for publication.

Methods:

1) **What were the criteria for discarding data (i.e. based on insufficient quality or quantity of data). As mixed models were used, were infants still included if they had data at least from some conditions, or was that infant removed entirely?**
The criteria for discarding data are described in section 2.3. When we first mention discarding participants in 2.1, we are now referring to 2.3 for technical details on the matter and are also specifying that discarding one participants means not including them in any of the analyses.
In the current version of the manuscript, we are also specifying the number of retained trials per condition.

2) **How was prosody type confirmed for stimuli in experiment 2?**
The stimuli used in Experiment 2 were developed by a different research group, who cured their validation as well. Besides citing the reference like in the previous version of our manuscript, we are now more clearly referring to their validation.

3) **Was experiment 1 always done first? If so, did infant fatigue/inattention influence results in experiment 2?**
Experiment order was counterbalanced across participants. We have specified this in the current version of the manuscript (Methods section). We are also reporting the actual number of participants in each group for which each order was administered.

4) **A silent movie was played during data acquisition, but what was this movie? If, for example, it involved social stimuli like faces, I think this would be a serious confounding factor.**
The movie (Inscapes) does not contain social stimuli, and has been validated in adults and children brain activity compared to total resting state. We have added the relevant reference to the movie.

5) **The authors do not explain why the temporal load sensors only were selected. Please add information about this.**
We have now specified the reason for scalp topography, and refer to limits of our probe in the Limitations.

6) **Why was gestational age not controlled for even though this was lower in the SMI-exposed group?**

Gestational age was not included as a covariate due to its moderate correlation with birth weight and to concerns on data quality. Of the two variables, birth weight was

considered the more reliably measured and less prone to recall or reporting bias, and was therefore retained in the analyses. We have now specified this more clearly in the Methods.

7) **Why were 8-10 months and 12 months in particular selected for assessment? Was 12 months a good choice considering that this is still a very early stage in terms of language development?**
The early time point in this study (9 months, range 8-10 months) was chosen in line with previous prospective studies in cohorts with elevated likelihood for atypical neurodevelopment. In the second half of the first year of life, infants are still pre-verbal, but their language comprehension is increasingly refined. Their preference for speech over non-speech is observed neurally and behaviourally, with some early signs of specialisation for their native language. We chose this time point to capture preference for speech at a pre-verbal stage, in order to consider this information in relation to later development.
The 12-month time point is also of particular interest, as it represents a key milestone in early language acquisition, including the emergence of first words and important precursors of expressive language.
The original project design included additional follow-up time points beyond 12 months. However, attrition rates after the 12-month assessment were too high to allow for reliable longitudinal analyses, and therefore the present study focuses on the data up to this time point.
We have now clarified these points and acknowledged the limitation in the revised manuscript.

Figures:

8) **In figures 1 and 2, please plot the individual data points for each infant**
We have added plots showing individual data points to the Supplementary Materials. While we agree that visualising individual data is important, we also believe that a concise display facilitates the overall interpretation of the results. We therefore opted for this solution to avoid overcrowding the main figures, as presenting individual data would require substantially more space (the two groups need separate plots for each time point and regression status).

Discussion:

9) **The evidence for SMI-exposed infants showing signs of language delay seems very weak, especially when considering the effects of SES as well. How strongly then can this be interpreted to be specific to SMI rather than SES?**

We agree that disentangling the effects of maternal mental illness and socio-economic status is challenging, and that SES may contribute to some of the observed differences in offspring outcomes.
The association between maternal mental illness and lower verbal IQ in offspring has been documented in the literature, although we acknowledge that our ability to fully replicate this pattern is limited by the relatively young age of our cohort. In our data, group differences in verbal IQ at 12 months were not conclusive, but they also did not clearly support the absence of a difference, which is remarkable at this early developmental stage.
Importantly, at 9 months, we observed group differences in fNIRS responses that remained significant even after accounting for SES. In addition, although signal properties at the channels showing group differences did not correlate with verbal IQ, such correlations were observed in adjacent channels (including within groups), suggesting some specificity in the observed effects.

Taken together, and in light of previous findings, we believe it is reasonable to interpret these early neural differences as primarily related to maternal diagnostic status. At the same time, we fully acknowledge the potential contribution of SES and have clarified this point in the Limitations, where we also remark how, at 12 months, it was not yet possible to replicate the expected group difference in verbal IQ.

10) **SMI mothers had lower SES- what are the authors thoughts about directionality of effects?**

This is indeed a very important point. Based on the present data, we cannot draw conclusions regarding the directionality of the association between SMI and lower SES.
On the one hand, severe mental health conditions may negatively impact educational attainment and occupational functioning, thereby contributing to lower SES. On the other hand, socioeconomic disadvantage – through factors such as financial strain, reduced access to education, and chronic stress – may increase the risk for the onset or exacerbation of mental health difficulties. Studies like the one by Mani et al. (2013) on poverty and cognitive performance in farmers suggest that a subject's IQ may vary over seasonal cycles together with their financial stability and could support this kind of directionality in broader contexts (i.e., SES affecting mental health, rather than vice-versa).
It is also plausible that this relationship is bidirectional, with mental health and SES influencing each other over time in a recursive manner.
In the Limitations section, where we note the SES difference between groups, we have also incorporated these considerations on directionality.

11) **Being able to record from only a small area of the scalp limits the conclusions that can be made about these findings (e.g. were responses specific to temporal areas, and if so/if not, what would this suggest). Please add some discussion on this issue.**
We have added this point in the Limitations section, as this is certainly a specific limitation of the fNIRS methodology, particularly used in infants, for whom the possibility to cover a large scalp area is particularly restricted.

Reviewer #3 (Remarks to the Author):

1) **Why did the authors focus on the bilateral temporal cortex and neglect the left prefrontal cortex, a region related with language closely?**
We agree that the left prefrontal cortex plays a key role in language processing and would be highly relevant to consider.
In the present study, our focus on bilateral temporal regions was both theoretically and methodologically motivated. From a theoretical perspective, temporal areas are critically involved in early auditory and speech processing, which are already well-established by around 9 months of age. In contrast, prefrontal regions—while important for higher-order language functions—are still undergoing substantial maturation during this developmental stage.
From a methodological standpoint, the fNIRS probe configuration available in our lab allowed for limited scalp coverage. We therefore prioritised bilateral temporal regions, given their central role in early-stage language and voice processing, which were the primary processes of interest in this study.
We have now clarified this rationale and explicitly acknowledged the limited spatial coverage, including the absence of prefrontal measurements, as a limitation in the manuscript.

2) **The authors should claim the reference points of the arrangement of fNIRS optode patches.**
The arrangement of the fNIRS optodes is based on the international 10–10 system. The corresponding reference points are reported in Table S1a in the Supplementary Materials, as the information is extensive and technical in nature.
In the revised manuscript, we have further clarified that the optodes were mounted using a standard, pre-perforated cap provided by the manufacturer, with locations reflecting the international 10–10 system (no lose patches were used, thus).

3) **To enhance readability, the authors could provide more details about the logic or reason supporting the series of data processing. The authors stated that the processing pipeline was adapted from the previous study. In this case, they were suggested to claim the adopted and adapted contents. Also, were these preprocessing methods contradict to each other?**
The processing steps were adapted from well-established and highly cited methodological papers. By "adapted," we meant that the overall pipeline and procedures were retained, while specific parameters (e.g., related to filtering and timing) were adjusted to match the characteristics of the current dataset, such as stimulus duration and the age of the participants.
In the revised manuscript, we changed the phrasing to avoid suggesting contradictions with published methodology.

4) **If possible, listing the valid fNIRS data for further analysis in each condition and experiment so that the readers could evaluate the soundness of the statistical analyses.**
The mean number of valid trials (with standard deviations) for each condition, group, and experiment was already reported in Section 2.3 of the original manuscript. As these values are highly similar across conditions and groups, we did not make further changes to this section.
We agree that providing this information is important for evaluating the robustness of the analyses, and we believe that the reported values already allow readers to assess the adequacy of the data included in the statistical models.

5) **Please claim the reason why GLM was not used to portrait the brain activity?**
While GLM approaches are widely used in fMRI, their application in fNIRS – particularly in infant populations – is less standardised and depends on the specific characteristics of the data and experimental design.
In the present study, we adopted a preprocessing and analysis pipeline based on established methodological work in infant fNIRS research. This approach relies on averaging haemodynamic responses across trials within condition, rather than fitting a GLM. This choice was motivated by several factors.
First, fNIRS signals provide a direct measure of haemodynamic changes with relatively high temporal resolution, such that condition-related HRFs can be characterised through block- or trial-averaging without the need for explicit model fitting.
Second, infant fNIRS data are characterised by substantial variability at the single-trial level, primarily due to motion artifacts and fluctuations in attention. While averaging across trials provides a robust estimate of the condition-specific response, this level of noise can reduce the stability of GLM-based estimates, especially when the number of valid trials per participant is limited.
Third, GLM approaches require assumptions about the shape and timing of the HRF. In infant populations, however, the haemodynamic response is known to show greater variability and developmental differences, making such assumptions less reliable and potentially introducing model misspecification.
Importantly, the adopted approach is widely used in infant fNIRS studies and is well-suited to capturing condition-related differences under these constraints.
We have now clarified this rationale in the manuscript.

6) **Further analysis targeting functional connectivity is also recommended.**
We thank the reviewer for this suggestion and agree that examining functional connectivity could provide important additional insights, particularly with respect to variables such as socio-economic status and maternal diagnosis.
However, we believe that functional connectivity analyses would be more appropriately conducted on resting-state data, which were not collected in the present study. In addition, our fNIRS setup was characterised by limited scalp coverage due to both technical constraints of the device and practical considerations related to infants' tolerance for the probe.
As a result, any connectivity analysis in the current dataset would be restricted to short-range connectivity or interhemispheric connectivity between bilateral temporal regions. While such analyses may be of general interest, they would not allow us to test the types of long-range connectivity patterns (e.g., fronto-temporal interactions) that are most likely to be influenced by maternal diagnosis and socioeconomic, as also highlighted by the Reviewer.
For these reasons, we feel that a functional connectivity analysis would be difficult to interpret meaningfully within the current dataset. We have now acknowledged this point in the Limitations and suggested it as an important direction for future research in the manuscript.
