# Supplementary material for: Early detection of distinct language development in infants of mothers with severe mental illness using fNIRS
Source: Commun Psychol. 2026 Jun 15;4:131. doi: 10.1038/s44271-026-00475-y (PMC13630572; doi:10.1038/s44271-026-00475-y)
Supplement: Supplementary file 2 [file 44271_2026_475_MOESM2_ESM.pdf]

Supplementary materials

S1

Source and detector optodes were placed at the 10-10 scalp locations listed below, forming 20 channels which, according to probabilistic estimates (Koessler et al., 2009) targeted the Brodmann areas indicated table S1a. Figure S1b represents the scalp location of sources and detectors, with the resulting channels and possible clusters.

Table S1a

Channels in the fNIRS probe, corresponding source-detector pairs, position of sources and detectors on the international 10-10 system, and targeted Brodmann areas based on probabilistic estimates.

Channel	Optodes	Scalp location (10-10 system)	Brodmann area
Ch01	S01-D01	FC5 – FT7	BA44L, BA47L
Ch02	S02-D01	T7 – FT7	BA42L, BA47L
Ch03	S01-D02	FC5 – C5	BA44L, BA42L
Ch04	S02-D02	T7 – C5	BA42L
Ch05	S03-D02	CP5 – C5	BA40L, BA42L
Ch06	S02-D03	T7 – TP7	BA42L, BA21L
Ch07	S03-D03	CP5 – TP7	BA40L, BA21L
Ch08	S04-D03	P7 – TP7	BA37L, BA21L
Ch09	S03-D04	CP5 – P5	BA40L, BA39L
Ch10	S04-D04	P7 – P5	BA37L, BA39L
Ch11	S05-D05	FC6 – FT8	BA44R, BA47R
Ch12	S06-D05	T8 – FT8	BA42R, BA47R
Ch13	S05-D06	FC6 – C6	BA44R, BA42R
Ch14	S06-D06	T8 – C6	BA42R
Ch15	S07-D06	CP6 – C6	BA40R, BA42R
Ch16	S06-D07	T8 – TP8	BA42R, BA21R
Ch17	S07-D07	CP6 – TP8	BA40R, BA21R
Ch18	S08-D07	P8 – TP8	BA37R, BA21R
Ch19	S07-D08	CP6 – P6	BA40R, BA39R
Ch20	S08-D08	P8 – P6	BA37R, BA39R

Figure S1b

Scalp position of sources, detectors, and channels in the probe.

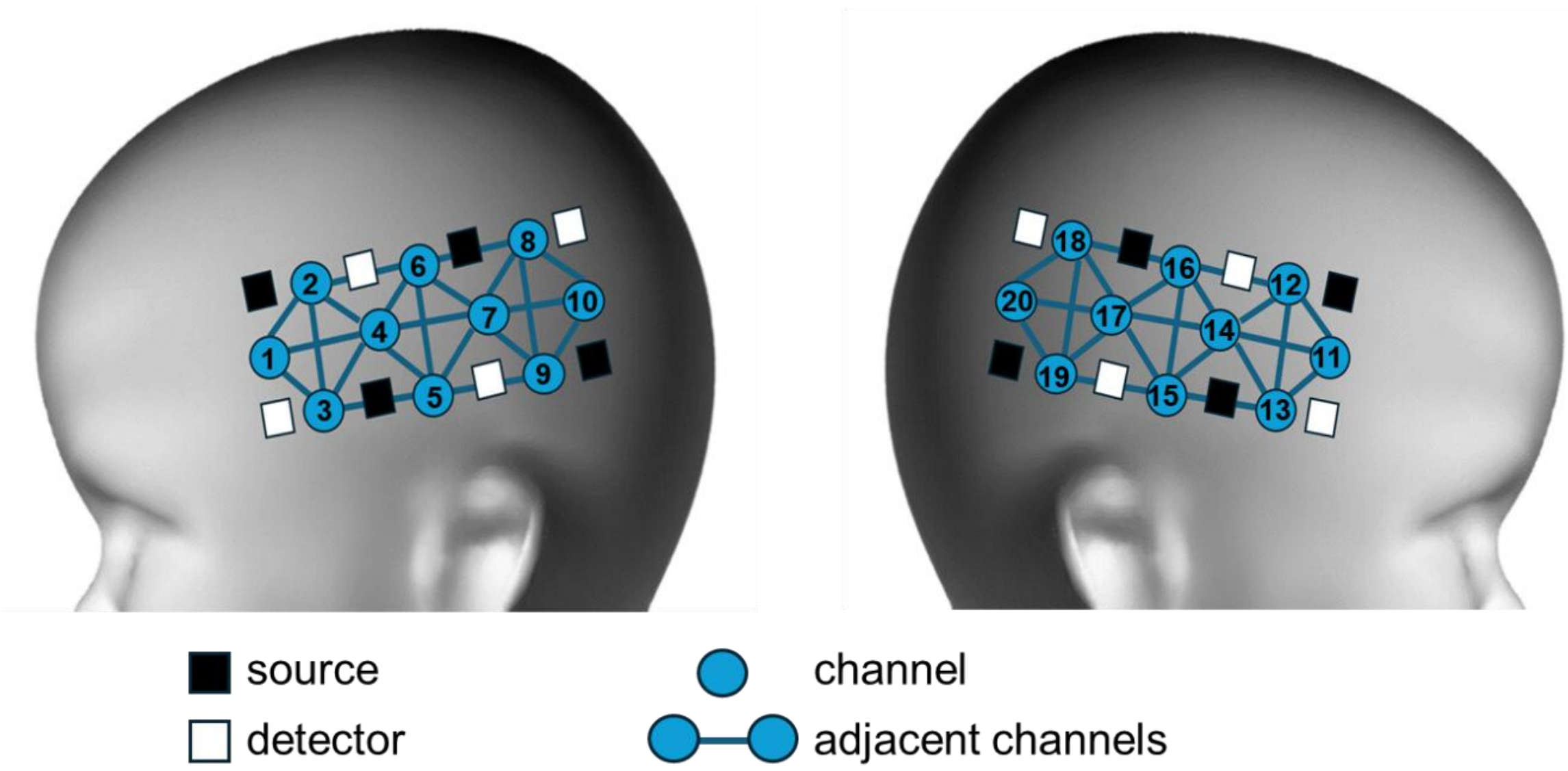

Note. Clusters include adjacent channels exceeding a predefined threshold; cluster-level statistics were tested against a permutation-based random distribution (Maris & Oostenveld, 2007).

Reference

Koessler, L., Maillard, L., Benhadid, A., Vignal, J. P., Felblinger, J., Vespignani, H., & Braun, M. (2009). Automated cortical projection of EEG sensors: anatomical correlation via the international 10-10 system. *NeuroImage*, *46*(1), 64–72. https://doi.org/10.1016/j.neuroimage.2009.02.006

S2.

The following materials illustrate HbO and Hbb concentration changes over the entire probe in response to voice and non-voice stimuli. S2a displays the results in exposed infants (n = 30) and S2b in controls (n = 30).

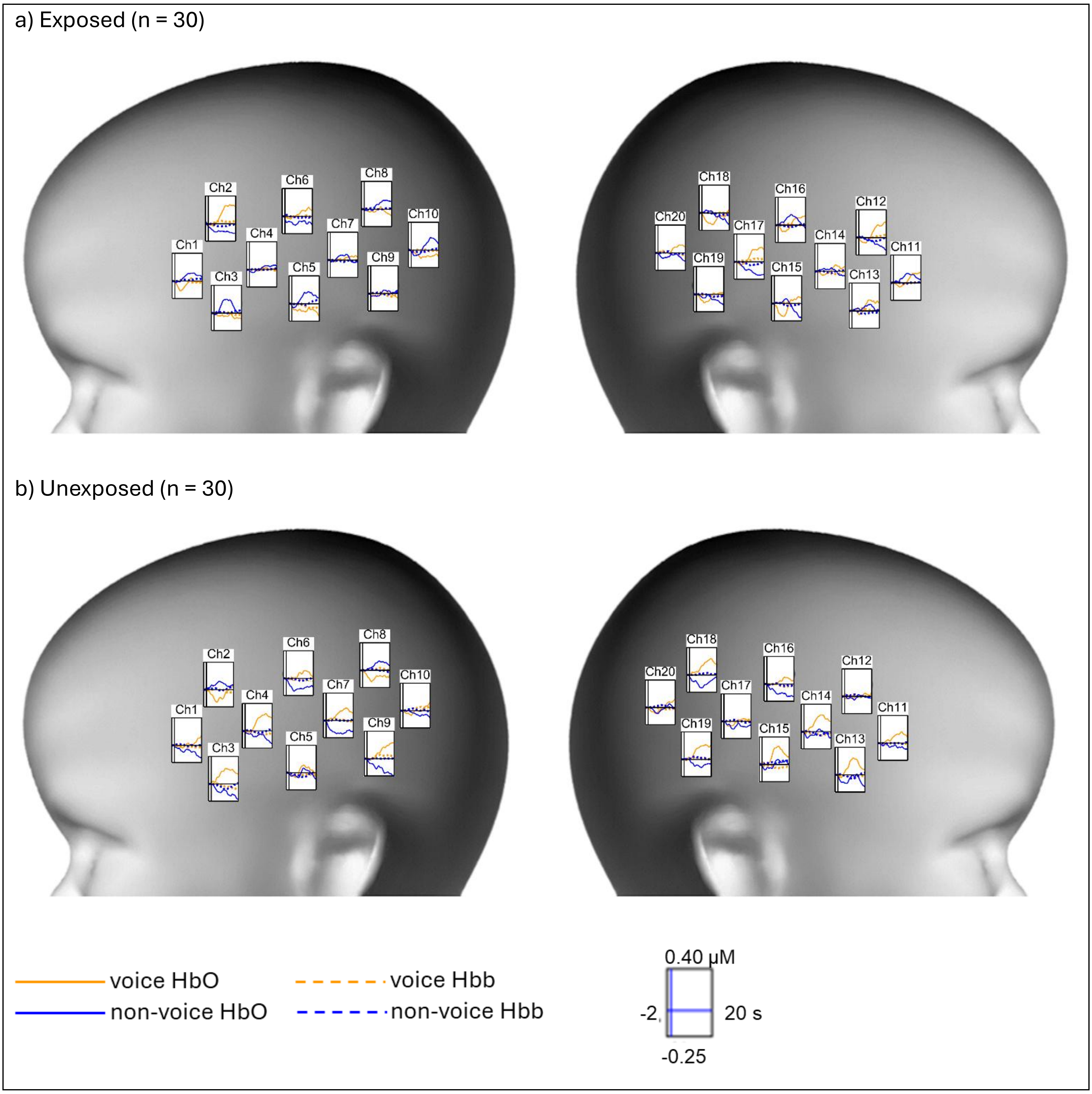

S3.

Figures S3a and S3b display HbO and Hbb concentration changes in response to sentences spoken with neutral prosody in exposed and unexposed infants, respectively. Figures S3c shows simple contrasts for emotional stimuli (angry minus neutral and happy minus neutral) in exposed infants (n = 30) and figure S3d represents the same contrasts for unexposed infants (n = 30).

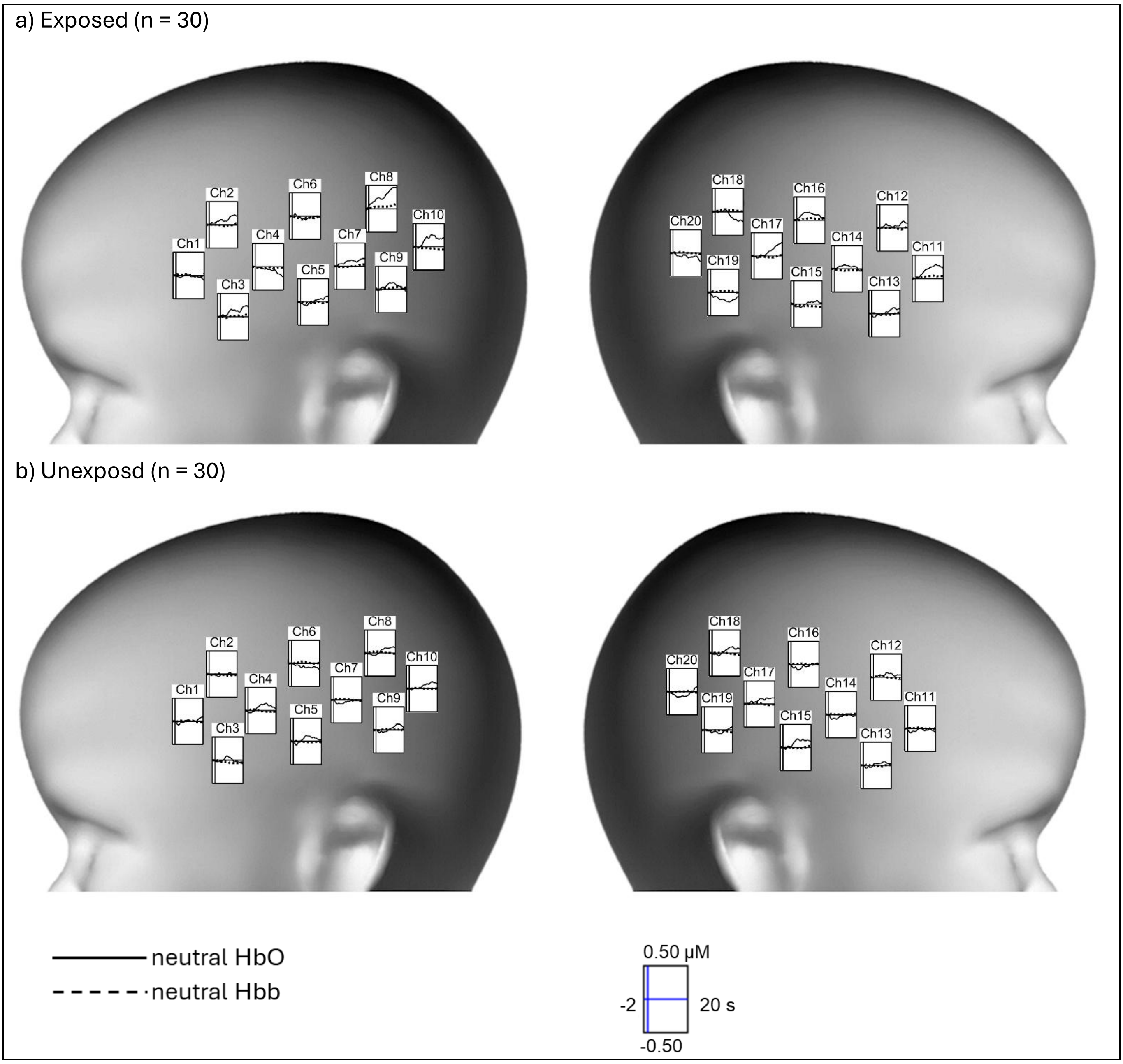

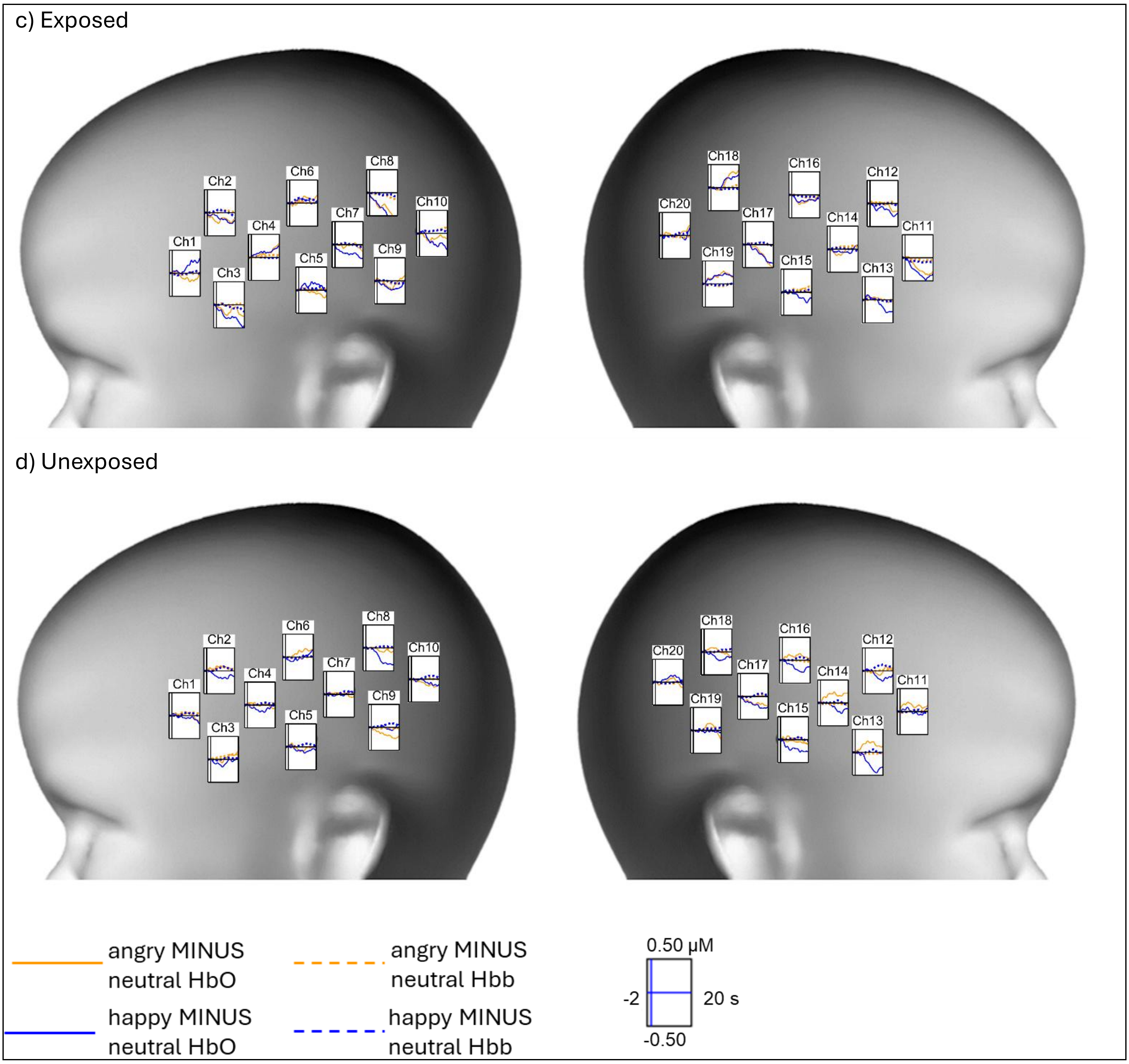
c) Exposed
Ch1
Ch2
Ch3
Ch4
Ch5
Ch6
Ch7
Ch8
Ch9
Ch10
Ch11
Ch12
Ch13
Ch14
Ch15
Ch16
Ch17
Ch18
Ch19
Ch20
d) Unexposed
Ch1
Ch2
Ch3
Ch4
Ch5
Ch6
Ch7
Ch8
Ch9
Ch10
Ch11
Ch12
Ch13
Ch14
Ch15
Ch16
Ch17
Ch18
Ch19
Ch20
angry MINUS neutral HbO
angry MINUS neutral Hbb
happy MINUS neutral HbO
happy MINUS neutral Hbb
0.50 μM
-2
20 s
-0.50
